# Proton Pump Inhibitor Prophylaxis After Gastric Bypass Does Not Cause Hypomagnesemia

**DOI:** 10.1007/s11695-015-2020-0

**Published:** 2016-01-14

**Authors:** Thomas C. C. Boerlage, Charlotte L. E. van Hees, Alwin D. R. Huitema, Fanny N. Lauw

**Affiliations:** Department of Internal Medicine, MC Slotervaart, Louwesweg 6, 1066 EC Amsterdam, The Netherlands; Department of Pharmacy & Pharmacology, The Netherlands Cancer Institute/MC Slotervaart, Plesmanlaan 121, Amsterdam, 1066 CX The Netherlands

**Keywords:** Proton pump inhibitor, Gastric bypass, Hypomagnesemia

## Abstract

Proton pump inhibitor (PPI)-induced hypomagnesemia is currently a major topic. Patients undergoing Roux-en-Y gastric bypass are generally prescribed PPI prophylaxis after surgery. We investigated the prevalence of hypomagnesemia in our bariatric population. We reviewed the files of 1000 postoperative patients for serum magnesium level during PPI use. We found only five cases of hypomagnesemia, none of which was evidently related to PPI use. We conclude that the risk of hypomagnesemia during 1 year of prophylactic PPI use after Roux-en-Y gastric bypass (RYGB) is minimal and laboratory screening is probably not necessary.

## Introduction

Hypomagnesemia following proton pump inhibitor (PPI) use was first reported in 2006 [1]. Several hundred cases have now been described [2]. A recent systematic review confirmed that PPI use significantly increases the risk of hypomagnesemia [3]. The relationship between the duration of PPI use and development of hypomagnesemia is unknown, but it is advised to monitor magnesium levels after more than 3 months of PPI use [4]. Other known risk factors for hypomagnesemia are diabetes mellitus, renal disease, age >65, and use of diuretics [3, 5]. Hypomagnesemia can lead to vomiting, diarrhea, tetany, seizures, QT-interval prolongation, and other electrolyte disturbances [2]. In most bariatric centers, patients are prescribed PPI prophylaxis after surgery. In our center, a PPI (pantoprazole 40 mg once daily) is prescribed to all patients for 1 year following surgery. After the first year, the PPI is continued only in specific cases, such as patients who suffered from a marginal ulcer. We investigated the prevalence of hypomagnesemia in our patient population.

## Methods

We reviewed the files of 1000 consecutive patients who underwent laparoscopic Roux-en-Y gastric bypass (RYGB) in our high-volume bariatric center between December 2012 and February 2014. Prior to surgery, patients underwent a screening program with laboratory testing, including serum magnesium levels. These same tests were routinely performed at 6 and 12 months after surgery. Patients receive lifelong supplementation with a daily multivitamin tablet, containing 125 mg magnesium.

Data were collected from the patient records. For this type of study, formal consent was not required. Serum magnesium level for all postoperative measurements was determined with the Architect ci8200 (Abbott diagnostics, IL, USA). Hypomagnesemia was defined as a magnesium level below the locally established lower reference value of 0.65 millimole per liter (mmol/L). In some of the cases, the preoperative magnesium level was determined with a different analyzer (Beckman Coulter LX-20 chemistry analyzer). This does not influence the results of this study as this concerns only preoperative measurements and the reference values are similar. However, a comparison of pre- and postoperative means could not be done.

## Results

Out of 1000 files studied, 931 patients had a magnesium level determined at either 6 or 12 months (214 patients) after surgery, or at both time points (717 patients). For 69 patients, there was no magnesium level available at both 6 and 12 months. Mean and standard deviation for each time point are shown in Table 1. Five patients had hypomagnesemia at 6 months, all suffered from diabetes (Table 2). Four of these patients already had hypomagnesemia preoperatively, two of them used a PPI preoperatively. Two patients had a normalized magnesium at 12 months without additional treatment despite continuing PPI use. One patient had a normal magnesium (0.67 mmol/L) preoperatively, despite already using a PPI at that time. This patient was 62 years old and suffered from diabetes mellitus type 2. The magnesium had decreased at 6 months (0.62 mmol/L) and 12 months (0.61 mmol/L). She was advised to discontinue PPI use.

**Table 1:** Magnesium levels at preoperative screening, 6 and 12 months

Time of measurement	Number of patients	Mean magnesium level (mmol/L)	Standard deviation (mmol/L)	Minimum and maximum levels (mmol/L)	Number of patients below reference value
Preoperative	983	0.825	0.070	0.56–1.08	11
6 months	862	0.839	0.064	0.55–1.05	5
12 months	783	0.848	0.066	0.61–1.09	2

*mmol*/*L* millimole per liter

**Table 2:** All patients with postoperative hypomagnesemia

Gender	Age (years)	Diabetes	Preoperative PPI use	Renal disease	Preoperative (mmol/L)	6 months (mmol/L)	12 months (mmol/L)
Male	44	Yes	No	Yes	0.56	0.55	0.61
Male	59	Yes	No	No	0.62	0.57	Unknown
Female	53	Yes	Yes	Yes	0.58	0.59	0.80
Female	53	Yes	No	No	0.58	0.62	0.69
Female	61	Yes	Yes	No	0.67	0.62	0.61

Lower reference value = 0.65 millimole per liter (mmol/L); renal disease = eGFR < 60

*PPI* proton pump inhibitor, *eGFR* estimated glomerular filtration rate

## Conclusion

In this retrospective study of 931 patients on prophylactic PPI after RYGB, we found no case of hypomagnesemia that was evidently related to the PPI use. This might be explained by the absence of other risk factors. Bariatric patients are relatively young, experience significant improvement in diabetes mellitus after surgery, and often discontinue the use of diuretics because of normalized blood pressure. Furthermore, the mean serum magnesium level is known to increase after RYGB, possibly due to improved insulin sensitivity or increased parathyroid hormone levels [6, 7].

We conclude that the risk of hypomagnesemia during 1 year of prophylactic PPI use after RYGB is minimal and laboratory screening is probably not necessary.
